# Shared atypical spontaneous brain activity pattern in early onset schizophrenia and autism spectrum disorders: evidence from cortical surface-based analysis

**DOI:** 10.1007/s00787-023-02333-2

**Published:** 2023-12-26

**Authors:** Xingyue Jin, Kun Zhang, Bin Lu, Xue Li, Chao-Gan Yan, Yasong Du, Yi Liu, Jianping Lu, Xuerong Luo, Xueping Gao, Jing Liu

**Affiliations:** 1 Department of Psychiatry, National Clinical Research Center for Mental Disorders, and National Center for Mental Disorders, The Second Xiangya Hospital of Central South University, Changsha, 410011 Hunan China; 2 CAS Key Laboratory of Behavioral Science, Institute of Psychology, Beijing, 100101 China; 3 Peking University Sixth Hospital, Peking University Institute of Mental Health, NHC Key Laboratory of Mental Health (Peking University), National Clinical Research Center for Mental Disorders (Peking University Sixth Hospital), 51 Huayuan Road, Haidian District, Beijing, 100191 China; 4 Shanghai Mental Health Center, No.600 Wanping Nan Road, Shanghai, China; 5 Department of Child Psychiatry of Shenzhen Kangning Hospital, Shenzhen Mental Health Center, Shenzhen, China; 6 Department of Psychology, University of Chinese Academy of Sciences, Beijing, 100101 China

**Keywords:** Schizophrenia, EOS, ASD, Autism, fMRI, ALFF, Cortex

## Abstract

Schizophrenia and autism spectrum disorders (ASD) were considered as two neurodevelopmental disorders and had shared clinical features. we hypothesized that they have some common atypical brain functions and the purpose of this study was to explored the shared brain spontaneous activity strength alterations in early onset schizophrenia (EOS) and ASD in the children and adolescents with a multi-center large-sample study. A total of 171 EOS patients (aged 14.25 ± 1.87), 188 ASD patients (aged 9.52 ± 5.13), and 107 healthy controls (aged 11.52 ± 2.82) had scanned with Resting-fMRI and analyzed surface-based amplitude of low-frequency fluctuations (ALFF). Results showed that both EOS and ASD had hypoactivity in the primary sensorimotor regions (bilateral primary and early visual cortex, left ventral visual stream, left primary auditory cortex) and hyperactivity in the high-order transmodal regions (bilateral SFL, bilateral DLPFC, right frontal eye fields), and bilateral thalamus. EOS had more severe abnormality than ASD. This study revealed shared functional abnormalities in the primary sensorimotor regions and the high-order transmodal regions in EOS and ASD, which provided neuroimaging evidence of common changes in EOS and ASD, and may help with better early recognition and precise treatment for EOS and ASD.

## Introduction

Schizophrenia (SCZ) and Autism spectrum disorders (ASD) have long been referred to as two neurodevelopmental disorders. Regarding the patients of the two diseases shared clinical features such as difficulties in social interaction and emotional reciprocity [1], it wasn’t until the Diagnostic and Statistical Manual of Mental Disorders (DSM)-III that autism was categorized as a pervasive developmental disorder rather than a psychosis. However, numerous studies in recent years have demonstrated overlapped abnormalities not only in symptoms but also in cognitive deficits [2, 3], genetic pathology [4, 5], structural and functional brain imaging alterations [6], which indicates more studies and evidence, especially in neurobiological pattern, are needed for better understanding of the pathological mechanism of SCZ and ASD.

The very earliest conceptualization of ASD described it as a specific subtype of SCZ, and now researchers still hold that autistic traits represent one of the important features of SCZ [7]. One noteworthy characteristic of both disorders is the abnormal sensory and perceptual function of the patients. While SCZ is characterized by verbal auditory hallucination, ASD patients may appear dysesthesia or hyperesthesia to pain, sound or light stimulus. Sensory-perceptual impairments exist not only as observable symptoms, but also were considered to be one of the key factors to social cognition deficit [8], which is another important issue in both disorders. Evidence from studies utilizing specific paradigms also showed that patients with SCZ or ASD had difficulty in visual tasks [9–11] which might contribute to their cognitive dysfunctions [11] and social impairments [12, 13], considering visual processing plays an important part in information processing and social interaction. As most ASD onset at an early age and social cognition is assumed as an early-developing process, it is reasonable to observe more severe social impairment in the ASD group. However, a meta-analysis showed no significant difference in social cognition between patients with SCZ or ASD at a mean age over 18 years old [14]. These similarities in dysfunction of sensory-perceptual and social cognition between SCZ and ASD suggest study on the underlying mechanism is meaningful, especially among patients under age 18 (schizophrenia patients who diagnosed before 18 years old called early onset schizophrenia, or EOS), given the developmental trajectory differences between SCZ and ASD. Considering spontaneous brain activity were essential in understanding these cognition variation [15], the intensity measurement of spontaneous neural activity — amplitude of low-frequency fluctuations (ALFF)—may add on imaging evidence of the similar dysfunctions on SCZ and ASD [16].

ALFF abnormalities in SCZ or in ASD vary across different brain regions. In SCZ patients, reduced ALFF in sensory and motor regions was observed [17], consistent with the clinical characteristics. Increased ALFF in the hippocampus and the left caudate of SCZ patients has been reported correlated with hallucinations [17, 18] and positive symptom scores [19]. In frontal regions, fractional ALFF (fALFF) decrease was related to poorer cognitive performance [15], while fALFF hyperactivation in this region, which has been suggested to associate with self-directed thoughts [17], as well as variation in the default mode network (DMN) leading to confusion between the source of internal thoughts and external perceptions, may contribute to the neurological basis for SCZ positive symptoms, including hallucinations [20]. Similarly, in posterior DMN, ASD patients exhibited significant lower ALFF values [21], which should be noted for its involvement in social cognitive processes; the researchers [21] also found in the follow-up cohort that the ALFF development from younger age to elder in ASD was different from that in typically developing children, based on which they projected that the atypical ALFF trajectory might be basis of ASD social defect, nevertheless, further studies were suggested. Based on previous evidence, researchers have proposed that the decrease of ALFF might be related to functional loss, while the increase of ALFF may manifest the compensation of maintaining general cognition level [22]. However, results of different studies remained controversial, and evidence from the adolescent population, which is significant for understanding neurodevelopmental disorders, are particularly insufficient. Most importantly, many studies have compared SCZ or ASD with healthy subjects, while a direct comparison of ALFF between SCZ and ASD, which may provide clearer evidences for the brain functional mechanism of these diseases, is missing.

In the present study, a multi-center large-sample study employing standardized functional magnetic resonance imaging (fMRI) sequence was designed to explore the brain spontaneous activity strength alterations in the children and adolescents with early onset schizophrenia (EOS) or ASD directly, as well as the similarities in EOS and ASD. Especially, we applied a surface-based analysis approach for fMRI data, rather than voxel-based analysis previous studies mostly used. The registration based on anatomy and the smoothing only including cortical surface of surface-based analysis makes is more accurate in showing information of brain sulcus and gyrus, and also more sensitivity than the volume-based analysis applied in most previous studies [23, 24]

## Methods

### Participants

In this cross-sectional multi-center designed study, participants were recruited through the outpatient clinic and advertisements and accepted resting-state fMRI scanning at mPeking University Sixth Hospital, Shanghai Mental Health Center, Shenzhen Kangning Hospital, The Second Xiangya Hospital of Central South University, and the East China Normal University.

The inclusion criteria for EOS individuals in the acute phase were: (1) aged 6–18 years old; (2) meet the criteria of SCZ according to the Diagnostic and Statistical Manual of Mental Disorders Fifth Edition (DSM-5); (3) first episode; (4) in the acute phase; (5) right handedness; (6) they can carry on the fMRI scanning. The exclusion criteria of the SCZ group were: (1) comorbidity with other severe psychiatric disorders such as major depressive disorder or bipolar disorder according to the Scheduled for Affective Disorders and Schizophrenia for School-Aged Children (K-SADS); (2) with clinical features of ASD; (3) with severe physical or neurological disease; (4) with metal implants in their body. The inclusion criteria for ASD individuals were: (1) aged 6–18 years old; (2) meet the criteria of ASD according to the Diagnostic and Statistical Manual of Mental Disorders Fifth Edition (DSM-5); (3) right handedness; (4) they can carry on the fMRI scanning. The exclusion criteria were: (1) comorbidity with other severe psychiatric disorders according to the Scheduled for Affective Disorders and Schizophrenia for School-Aged Children (K-SADS); (2) with severe physical or neurological disease; (3) with metal implants in their body. The criteria for health control participants (HC) were: (1) aged 6–18 years old; (2) without psychiatric disorders according to the K-SADS; (3) without neurodevelopment diseases such as ASD; (4) without a family history of psychiatric disorders; (5) right handedness; (6) they can carry on the fMRI scanning. The exclusion criteria were: (1) comorbidity with severe physical or neurological disease; (2) with metal implants in their body. All participants were informed and signed informed consent by themselves and (or) their guardians. This study was approved by the Ethics Committee of Peking University Sixth Hospital.

### Image acquisition

All participants received resting-state fMRI scanning in 5 different scanners: (1) 3 Tesla GE MR750 scanner at Peking University Sixth Hospital, (2) 3 Tesla SIEMENS Skyra scanner at The Second Xiangya Hospital of Central South University, (3) 3 Tesla GE MR750 scanner at Shenzhen Kangning Hospital, (4) 3 Tesla SIEMENS Prisma scanner at East China Normal University and (5) 3 Tesla SIEMENS Verio scanner at Shanghai Mental Health Center.

Participants were instructed to lie still in the scanner with a relaxed state and eyes closed. Earmuff was used to reduce noise interference. The scanning protocols for 3D T1-weighted image and BOLD (blood oxygen level dependent) resting-state functional image among these five scanners were standardized to minimize the side effect by Chinese Association of Brain Imaging. The major parameters were kept consistent among these scanners. The scanning protocol for 3D T1-weighted imaging included: slice number = 192, matrix size = 256 × 256, FOV = 256 × 256 mm, TR/TE/TI = 2530/2.96/1100 ms (SIEMENS), 6.7/2.9/450 ms (GE), FA = 7° (SIEMENS), 12° (GE), slice thickness = 1 mm, gap = 0, voxel size = 1 × 1 × 1 mm, iPAT = 2/32 (SIEMENS), Aset = 2 (GE). The scanning protocol for BOLD resting-state functional imaging included: slice number = 43, matrix size = 64 × 64, FOV = 220 × 220 mm, TR/TE = 2000/30 ms, FA = 90°, slice thickness = 3.2 mm, gap = 0, voxel size = 3.4 × 3.4 × 3.2 mm, number of acquisitions = 240, NEX = 1, parallel acceleration = 2.

### MRI progressing

Data preprocessing was performed by DPABISurf [25], a surface-based fMRI data analysis toolbox evolved from DPABI/DPARSF. DPABISurf used docker technology to wrap the whole computing environment for fMRIPrep [26], FreeSurfer [27], ANTs [28], FSL [29], PALM [30] and et al. The preprocessing procedure includes: (1) Converting images to NIFTI format and BIDS format; (2) intensity nonuniformity correction and skull-stripping; (3) tissue segmentation of cerebrospinal fluid (CSF), white matter (WM) and gray matter (GM); (4) brain surface reconstruction; (5) deleting first ten time points; (6) boundary-based registration; (7) slice-timing correction; (8) normalization to fsaverage5 space; (9) head motion, WM and CSF signal and linear trend nuisance regression; (10) bandpass filtering (0.01–0.1 Hz); (11) spatial smoothing (full­width at half­maximum (FWHM) of 6 mm). Data with bad acquisition or inaccurate reconstruction were manually excluded from quality control and participants whose head motion (mean- FD-Jenkinson) exceeded 0.2 mm were excluded too. After that, ALFF was computed for each subject using DPABISurf at a 0.01–0.08 Hz frequency band.

### Statistical analyses

Group differences of demographics between EOS, ASD and HC were compared with Kruskal–Wallis test and Chi-square test, post-hoc analyses with Bonferroni corrected were used to examine the differences between groups. After getting the ALFF data in the cortex and subcortical area, analysis of covariance analysis (ANCOVA) was used to calculate the ALFF difference among the SCZ, the ASD and the HC group. Age, sex, and head motion (mean-FD-Jenkinson) were included as covariates in the general linear model. ComBat was used to control potential site and scanner biases [31]. The statistical maps of ANCOVA were corrected for family-wise error rate using Gaussian random field (GRF). The vertex-wise threshold was 0.001 and the cluster-wise threshold for GRF correction was 0.017 (0.05/3, 3 for Bonferroni correction of two hemispheres and subcortex). To get specific differences between the EOS and HC group, the ASD and HC group, and between the ASD and EOS group, we extracted the ALFF data in the brain regions that showed differences in ANCOVA and carried out a post-hoc two-sample t test to compare every two groups.

## Results

In this study, 666 participants were invited and received resting-state fMRI scanning, after excluded 200individuals with bad images acquisition quality, inaccurate reconstruction or huge head motion (mean FD-Jenkinson exceeding 0.2 mm), a total of 466 participants including 171 participants with EOS, 188 participants with ASD, and 107 participants in the HC group were included in the following analysis, 10 EOS and 20 ASD had received psychiatric medication (Table 1). Specific information about these five sites were listed in Table 2.

**Table 1:** The demographic information of the participants

	EOS (*n* = 171)	ASD (*n* = 188)	HC (*n* = 107)	*H*/Chi^2^	*p*
Age (mean ± SD)	14.25 ± 1.87	9.52 ± 5.13	11.52 ± 2.82	22.549	< 0.001^a,b^
Sex (male/female)	66/105	156/32	62/45	98.403	< 0.001^a,b,c^
Head motion(mean ± SD)	0.063 ± 0.040	0.075 ± 0.041	0.080 ± 0.036	124.329	< 0.001^a,b^
Psychiatric drug use (yes/no)	10/161	20/168	0/107	9.180	0.010^b,c^

The head-motion values listed in the table are the mean FD-Jenkinson values of the participants. After Bonferroni correction, significant group differences were marked as: ^a^between EOS and ASD, ^b^between EOS and HC, ^c^between ASD and HC

EOS, early onset schizophrenia; ASD, autism spectrum disorder; HC, healthy control; SD, standard deviation

**Table 2:** The site information

	Site 1 (*n* = 311)	Site 2 (*n* = 100)	Site 3 (*n* = 136)	Site 4 (*n* = 104)	Site 5 (*n* = 15)
Sex(male/female)	100/211	53/47	33/103	56/48	8/7
Group(EOS/ASD/HC)	50/171/90	80/20/0	18/100/4/	75/6/23	7/8/0
Psychiatric drug use (yes/no)	4/307	18/82	133/3	99/5	0/15
Age (mean ± SD)	12.47 ± 4.04	14.11 ± 2.10	6.81 ± 4.41	13.81 ± 1.99	13.80 ± 2.24

Site 1, Peking University Sixth Hospital; Site 2, Shanghai Mental Health Center; Site 3, Shenzhen Kangning Hospital. Site 4, The Second Xiangya Hospital of Central South University; Site 5, the East China Normal University

EOS, early onset schizophrenia; ASD, autism spectrum disorder; HC, healthy control; SD, standard deviation

**Fig. 1:**
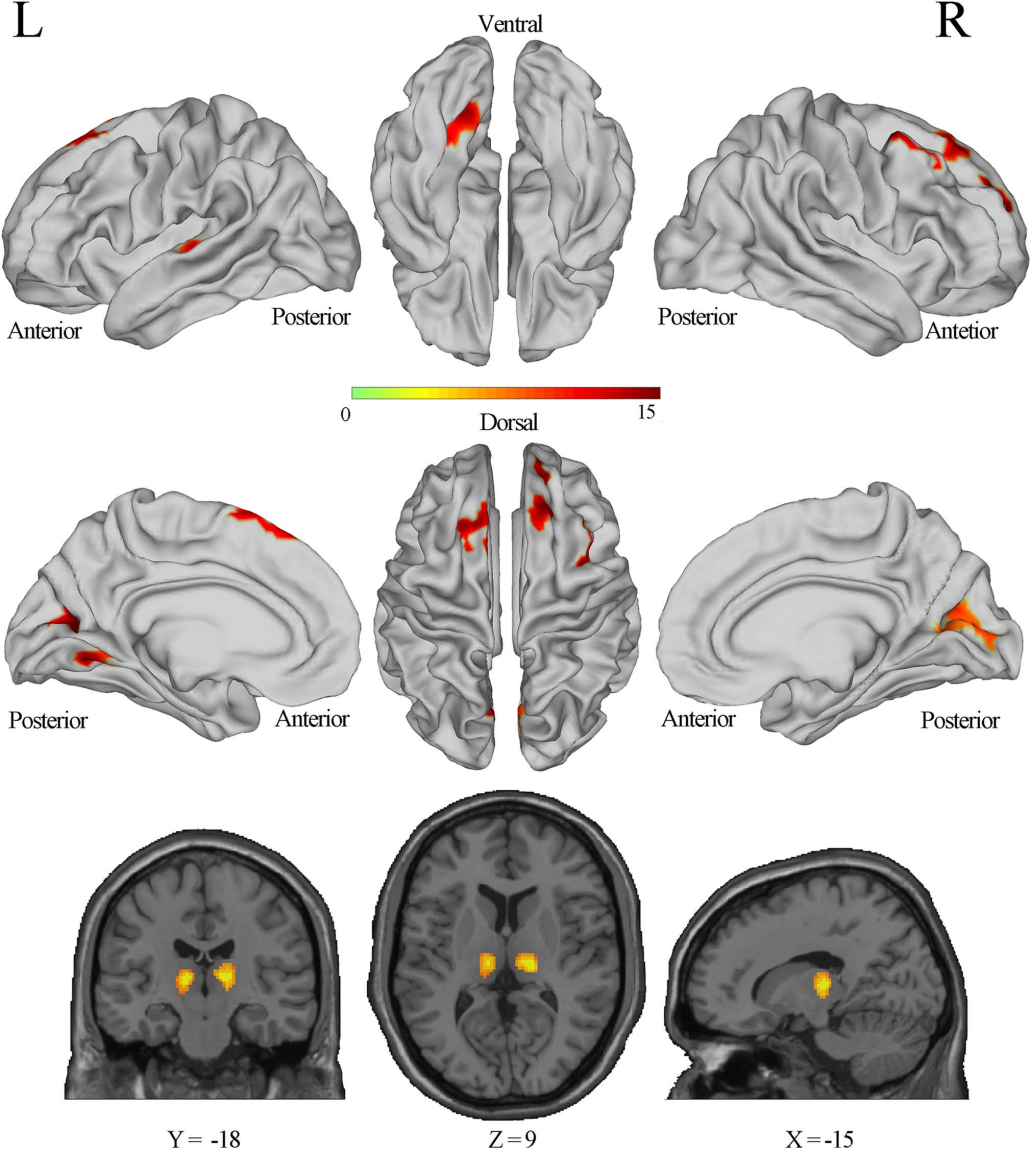
Brain areas with significant differences in ALFF among the EOS, ASD, and HC groups

The ANCOVA showed a total of 10 clusters met the vertex-wise threshold < 0.001 and the cluster-wise threshold for GRF correction < 0.017 and were significantly differenced among three groups (Fig. 1, Table 3). For the cortical areas, bilateral superior frontal language area (SFL), bilateral dorsolateral prefrontal cortex (DLPFC), bilateral primary visual cortex, bilateral early visual cortex, left ventral visual stream, and left early auditory cortex (including primary auditory cortex [PAC], lateral belt, parabelt), and right frontal eye fields showed differences among the groups. These areas are located in DMN, visual network (VN), somatomotor network (SMN), and frontoparietal network (FPN) according to the large-scale functional network [32]. For the subcortical areas, bilateral thalamus showed significant differences among the groups in the volume space.

**Table 3:** Brain areas with significant differences in ALFF among the EOS, ASD, and HC groups

No	Hemisphere	Cluster size (mm^2^)	Brain region	Network	Peak intensity (*F*)	Peak coordinate			Post-hoc (t)		
						*X*	*Y*	*Z*	ASD-HC	EOS-HC	ASD-EOS
1	L1	467	Primary visual cortex Early visual cortex	Visual	9.27	26.1	− 80.8	− 3.3	− 2.21*	− 4.63***	2.62**
2	L2	522	Ventral visual stream Primary visual cortex Early visual cortex	Visual	12.20	14.0	− 57.1	− 35.8	− 1.13	− 5.22***	3.81***
3	L3	520	SFL, DLPFC, Paracentral lobular	Default	10.47	16.1	56.7	51.0	0.84	5.18***	− 3.18***
4	L4	209	PAC, lateral belt, parabelt	Somatomotor	12.96	− 28.9	− 6.2	− 15.8	− 4.05***	− 4.25***	0.65
5	R1	217	DLPFC, frontal eye fields	Frontoparietal	9.79	5.2	33.0	55.1	2.49**	5.23***	− 3.04**
6	R2	382	DLPFC, SFL	Default	12.36	− 21.3	37.4	29.7	2.55**	5.69***	− 2.86**
7	R3	216	DLPFC	Default	8.83	− 19.4	65.5	7.8	3.08**	5.25***	− 1.44
8	R4	973	Primary visual vortex Early visual cortex	Visual	10.61	− 30.8	− 61.1	− 22.1	− 3.61***	− 4.69***	1.14
9	V1 L	213	Thalamus	-	16.33	− 11.5	− 18.5	7.5	− 3.04**	− 4.69***	1.74*
10	V2 R	178	Thalamus	-	14.55	14.5	− 20.5	7.5	− 2.90**	− 4.82***	1.73

ALFF, amplitude of low-frequency fluctuations; EOS, early onset schizophrenia; ASD, autism spectrum disorder; HC, healthy control; SFL, superior frontal language area; DLPFC, dorsolateral prefrontal cortex; PAC, primary auditory cortex

^*^p < 0.05; **p < 0.01; ***p < 0.001

Post-hoc analysis showed that both participants with EOS or with ASD had significant higher ALFFs in right frontal eye fields, right premotor cortex, right SFL and right DLPFC than the HC group. And both participants with EOS or with ASD showed a significantly lower ALFF in bilateral primary and early visual cortex, left early auditory cortex than the HC group, which are located in VN and SMN. The ALFF of the left ventral visual stream was also significantly lower in EOS than in HC, but the ASD group showed no significant difference from the HC group in this region. Moreover, compared with participants with EOS, participants with ASD had significantly higher ALFF in the left primary and early visual cortex, and left ventral visual stream. ASD also had lower ALFF in bilateral SFL and bilateral DLPFC, right frontal eye fields, and right premotor cortex than EOS. As for the subcortex areas, both ASD and EOS showed a significantly lower ALFF in bilateral thalamus than HC, and EOS had greater changes than ASD.

In summary, EOS or ASD patients showed higher ALFF than healthy controls (in a descending order of EOS > ASD > HC) in the transmodal networks such as DMN. On the contrary, EOS or ASD patients showed lower ALFF than healthy controls (in an ascending order of EOS < ASD < HC) in the primary sensory/motor networks such as VN and SMN. These findings indicate that the ALFF abnormalities exhibit entirely opposing trends at the two ends of the functional hierarchy, often referred to recently as the first functional gradient [33]. When compared with ASD patients, EOS patients consistently demonstrated more pronounced abnormalities in spontaneous brain activity intensity.

## Discussion

In this study, a shared atypical spontaneous brain activity pattern in functional hierarchy including hypoactivity in the primary sensorimotor regions (e.g., VN and SMN) and hyperactivity in the high-order transmodal regions (e.g., DMN) was found in both EOS and ASD groups. Importantly, the EOS group showed more severe abnormality in the both end of the functional hierarchy compared with ASD group. To the best of our knowledge, this is the first study to examine altered brain spontaneous activity intensity in both EOS, ASD, and HC adolescents in the brain surface.

Recent studies hypothesized that both SCZ and ASD are in the model of a neurodevelopmental continuum, share genetic risk and pathogenic mechanisms and represent diverse brain development outcomes [34]. SCZ and ASD are reframed as bodily self-consciousness [35] and lead to phenotypic overlap as social communication impairments [36, 37].

A series of important, common and characteristic symptoms in SCZ are auditory hallucinations, which are perceptional experiences of voices that occur without external stimulus [38]. Previous studies showed that patients with SCZ had no significant deficits in routine hearing tests, but typically exhibited high-order auditory deficits such as higher threshold in tone-match tasks [39, 40], indicating dysfunction in brain auditory regions. Compared with HC, patients with recent-onset SCZ showed decreased functional connectivity (FC) in the auditory networks [41]. Among auditory cortex regions, Heschl’s gyrus (HG) was found to be associated with SCZ auditory hallucinations. Compared with SCZ patients without auditory hallucinations, SCZ patients with auditory hallucinations showed a significantly thinner cortex in the left HG [42]. Patients with SCZ also showed altered functional asymmetry in HG in resting state, these changes are correlated with their acute positive symptoms [43]. In addition, the transverse gyrus of HG contains part of the primary auditory cortex (PAC) which plays a critical role in early auditory processing [44]. Primary auditory cortex (PAC) dysregulation could give rise to auditory verbal hallucinations due to being overly sensitive to internal processing activation, and less responsive to external stimulation [45]. Meanwhile, researchers also found auditory verbal hallucination-related activation in the PAC regions [46], and SCZ patients with auditory hallucinations showed significantly increased co-activation in the right auditory cortex and bilateral insula within the auditory network compared with those without auditory hallucinations [47]. In the present study, we found that adolescents with SCZ showed lower ALFF in the PAC, lateral belt and parabelt which combined as early auditory cortex [48], based on previous evidence, we projected that lower ALFF in the early auditory cortex might be associated with auditory hallucinations in adolescents with SCZ.

In ASD, we found lower ALFF in the bilateral primary and early visual cortices and the left early auditory cortex. It has been proposed that altered sensory processing may cause core features of autism [49]. In visual domain, ASD patients often showed abnormal visual preferences, they obtained details of the perceptual world and ignore the global percept [50]. Some of the patients exhibit atypical visual behaviors such as attempting to avoid visual input (e.g., covering eyes at bright light) or to seek additional visual stimuli (e.g., twisting fingers in front of eyes) [51]. Regarding sensory processing deficits in ASD, previous studies revealed that ASD patients had significantly weaker FC between the visual cortex and sensorimotor regions, and the weaker FC was correlated with increased sensory hypersensitivity in the visual and auditory domains [52]. However, Chen et al. had controversial findings showing increased intrinsic FC between visual and sensorimotor networks in ASD, and the overconnectivity was more marked in those with more severe symptoms [53]. In auditory domain, ASD patients showed enhanced pitch discrimination and the increased auditory perception may partly explain the auditory superiorities such as heightened pitch detection in ASD, yet which subsequently can interfere with social communication.[54]. Millin et al. found ASD patients had abnormal activation in the auditory cortex during the repeated audio–visual stimulation task, which reflected reduced adaption to audio stimulation, and the degree of activation in auditory cortex was correlated with ASD symptom severity [55]. Their findings might be explained by abnormality in information-processing of ASD patients [56], which is associated with behavioral traits such as aversion to environmental sounds. Few studies have involved surface-based analysis of ALFF in ASD. Our findings added evidence to the abnormal brain function of visual and auditory domains in ASD.

DMN activation is associated with individual internal activities, such as autobiographical memory retrieval and conceiving the perspectives of others [57], leading to its involvement in social cognitive processes [58]. We have found increased ALFF in the area of SFL and DLPFC in both EOS and ASD, which were important parts of the DMN [32]. SCZ and ASD share marked social deficits [58], thus we hypothesized that the overactive of DMN, reflecting undue individual internal activities, might be associated with social deficits in adolescents with SCZ or ASD. The mPFC plays an important role in the cognitive process, emotional regulation, socializing and motivation, the alterations of the neuronal excitation and inhibition balance in mPFC can partly explain the similar social deficits among SCZ and ASD [59]. In adult patients with SCZ, ALFF reduction was found in the left ACC [60]; while increased FC between DMN and central executive network was related with hallucinations severity [61]. Chen et al. found the FC involving the DMN were decreased in adolescents with SCZ or ASD [62], however, they didn’t compare the SCZ group with the ASD group directly. While previous studies showed controversial results, overall, different DMN connectivity pattern was found between SCZ and ASD, with more studies showing overconnected in SCZ and under-connected in ASD [63]. These differences may be due to the diversity of developmental trajectory of participants at different age [21, 64]. Thus, neural development abnormality may also affect the brain functional activities of patients at different ages. In addition, the difference may be explained by different analysis methods and sample sizes [65].

Our findings should be interpreted with caution in light of several limitations. First, the modeling of low-frequency (0.01–0.1 Hz) fluctuations of the BOLD signal used in the present study reduced but could not eliminate the aliased physiological noise such as cardiac and respiratory noises [66]. Second, there was a mismatch in age and sex among EOS, ASD and the HC group. Third, the strict quality control of head motion during fMRI data progress may exclude patients with severer symptoms, which lowers the representativeness of this study. Moreover, the potential effect of drug use on the findings cannot be ruled out, future studies should be involved in more drug naïve individuals.

In conclusion, this study revealed shared functional abnormalities in the primary sensorimotor regions and the high-order transmodal regions in EOS and ASD. EOS had overall larger changes than ASD in the shared abnormalities. This study provided valuable evidence of the pathological connections between EOS and ASD regarding surface-based fMRI analysis. It highlighted the similarities between these two diseases, and differences with healthy participants, which might help with the early recognition of EOS and ASD, and may also provide some foundations of precise treatment for schizophrenia and ASD.

## Data Availability

The data that support the findings of this study are available on request from the corresponding author. The data are not publicly available due to privacy or ethical restrictions.
