# Correction to: Shared atypical spontaneous brain activity pattern in early onset schizophrenia and autism spectrum disorders: evidence from cortical surface-based analysis

**DOI:** 10.1007/s00787-024-02409-7

**Published:** 2024-03-06

**Authors:** Xingyue Jin, Kun Zhang, Bin Lu, Xue Li, Chao-Gan Yan, Yasong Du, Yi Liu, Jianping Lu, Xuerong Luo, Xueping Gao, Jing Liu

**Affiliations:** 1 Department of Psychiatry, National Clinical Research Center for Mental Disorders, and National Center for Mental Disorders, The Second Xiangya Hospital of Central South University, Changsha, 410011 Hunan China; 2 CAS Key Laboratory of Behavioral Science, Institute of Psychology, Beijing, 100101 China; 3 Department of Psychology, University of Chinese Academy of Sciences, Beijing, 100101 China; 4 Peking University Sixth Hospital, Peking University Institute of Mental Health, NHC Key Laboratory of Mental Health (Peking University), National Clinical Research Center for Mental Disorders (Peking University Sixth Hospital), 51 Huayuan Road, Haidian District, Beijing, 100191 China; 5 Shanghai Mental Health Center, No.600 Wanping Nan Road, Shanghai, China; 6 Department of Child Psychiatry of Shenzhen Kangning Hospital, Shenzhen Mental Health Center, Shenzhen, China

**Correction to: European Child and Adolescent Psychiatry** 10.1007/s00787-023-02333-2

In this article the affiliation details for Author Bin Lu, Chao-Gan Yan were incorrectly given as ‘Peking University Sixth Hospital, Peking University Institute of Mental Health, NHC Key Laboratory of Mental Health (Peking University), National Clinical Research Center for Mental Disorders (Peking University Sixth Hospital), 51 Huayuan Road, Haidian District, Beijing 100191, China’ but should have been ‘CAS Key Laboratory of Behavioral Science, Institute of Psychology, Beijing 100101, China’.

The affiliation details for Author Xue Li and Jing Liu were incorrectly given as ‘CAS Key Laboratory of Behavioral Science, Institute of Psychology, Beijing 100101, China’ but should have been ‘Peking University Sixth Hospital, Peking University Institute of Mental Health, NHC Key Laboratory of Mental Health (Peking University), National Clinical Research Center for Mental Disorders (Peking University Sixth Hospital), 51 Huayuan Road, Haidian District, Beijing, 100191, China’.

The original article has been corrected.

